# Unlocking the potential of photon counting detector CT for paediatric imaging: a pictorial essay

**DOI:** 10.1093/bjro/tzae015

**Published:** 2024-07-09

**Authors:** Ieva Aliukonyte, Daan Caudri, Ronald Booij, Marcel van Straten, Marcel L Dijkshoorn, Ricardo P J Budde, Edwin H G Oei, Luca Saba, Harm A W M Tiddens, Pierluigi Ciet

**Affiliations:** Department of Radiology and Nuclear Medicine, Erasmus MC—Sophia Children’s Hospital, Rotterdam, 3015 GD, The Netherlands; Department of Paediatric Pulmonology and Allergology, Erasmus MC—Sophia Children’s Hospital, Rotterdam, 3015 GD, The Netherlands; Department of Radiology and Nuclear Medicine, Erasmus MC—Sophia Children’s Hospital, Rotterdam, 3015 GD, The Netherlands; Department of Paediatric Pulmonology and Allergology, Erasmus MC—Sophia Children’s Hospital, Rotterdam, 3015 GD, The Netherlands; Department of Radiology and Nuclear Medicine, Erasmus MC—Sophia Children’s Hospital, Rotterdam, 3015 GD, The Netherlands; Center for Medical Image Science and Visualization (CMIV), Linköping University, Linköping, SE-581 83, Sweden; Department of Radiology and Nuclear Medicine, Erasmus MC—Sophia Children’s Hospital, Rotterdam, 3015 GD, The Netherlands; Department of Radiology and Nuclear Medicine, Erasmus MC—Sophia Children’s Hospital, Rotterdam, 3015 GD, The Netherlands; Department of Radiology and Nuclear Medicine, Erasmus MC—Sophia Children’s Hospital, Rotterdam, 3015 GD, The Netherlands; Department of Radiology and Nuclear Medicine, Erasmus MC—Sophia Children’s Hospital, Rotterdam, 3015 GD, The Netherlands; Department of Radiology of Cagliari, Polo di Monserrato—Azienda Ospedaliero Universitaria (A.O.U.), Cagliari, 09042, Italy; Department of Radiology and Nuclear Medicine, Erasmus MC—Sophia Children’s Hospital, Rotterdam, 3015 GD, The Netherlands; Department of Paediatric Pulmonology and Allergology, Erasmus MC—Sophia Children’s Hospital, Rotterdam, 3015 GD, The Netherlands; Department of Radiology and Nuclear Medicine, Erasmus MC—Sophia Children’s Hospital, Rotterdam, 3015 GD, The Netherlands; Department of Paediatric Pulmonology and Allergology, Erasmus MC—Sophia Children’s Hospital, Rotterdam, 3015 GD, The Netherlands; Department of Radiology of Cagliari, Polo di Monserrato—Azienda Ospedaliero Universitaria (A.O.U.), Cagliari, 09042, Italy

**Keywords:** photon counting CT, paediatric imaging, imaging techniques

## Abstract

Recent advancements in CT technology have introduced a revolutionary innovation to practice known as the Photon-Counting detector (PCD) CT imaging. The pivotal hardware enhancement of the PCD-CT scanner lies in its detectors, which consist of smaller pixels than standard detectors and allow direct conversion of individual X-rays to electrical signals. As a result, CT images are reconstructed at higher spatial resolution (as low as 0.2 mm) and reduced overall noise, at no expense of an increased radiation dose. These features are crucial for paediatric imaging, especially for infants and young children, where anatomical structures are notably smaller than in adults and in whom keeping dose as low as possible is especially relevant. Since January 2022, our hospital has had the opportunity to work with PCD-CT technology for paediatric imaging. This pictorial review will showcase clinical examples of PCD-CT imaging in children. The aim of this pictorial review is to outline the potential paediatric applications of PCD-CT across different anatomical regions, as well as to discuss the benefits in utilizing PCD-CT in comparison to conventional standard energy integrating detector CT.

## Introduction

An advancement in CT technology has been recently achieved by the introduction of Photon-Counting detector (PCD) scanners. The revolutionary aspect of this technology lies in its novel detectors. PCDs contain smaller pixels than the standard detectors used in energy integrating detector CT scanners (EID-CT) and they allow a direct conversion of X-rays into an electrical signal.^1^ Most clinical CT scanners operate with EIDs, which have seen only limited reduction in pixel size over the last decade, and thus not much improvement in the spatial resolution.^1^ This is due to the current geometry of EID elements, which necessitate thin reflecting septa to prevent cross-talk between light photons generated in the scintillator.^1^ During cross-talk, the signals scattering from one detector element interfere with the signals from adjacent elements, leading to errors or artefacts in the CT image.^1^ Conversely, PCDs do not use scintillators and eliminate the need of septa between detector elements. Instead, PCDs directly convert X-ray photons into electrical signals. PCDs are made of a single semiconductor diode layer,^1^ which thanks to the direct conversion of X-ray photons into an electrical signal can determine the energy level for each detected X-ray photon (spectrum).^1^ While PCDs can sort different X-ray photons based on energy level and exclude electronic noise using specific threshold levels, EIDs only measure the total energy deposited, including electronic noise.^1^ The hardware improvements of PCD-technology lead to several advantages, including lower electronic noise, increased iodine signal-to-noise ratio (SNR), improved spatial resolution and SNR, reduced radiation dose and improved visualization of small anatomical structures.^2^

These improved parameters are crucial in all imaging, but of particular importance in paediatric imaging, where radiation dose should always be minimized in accordance with the image gently campaign.^3^ The increased SNR of PCD-CT allows dose reduction without compromising image resolution, or maintaining the same dose as EID-CT while increasing spatial resolution. The latter is pivotal for children under 5 years of age where anatomical structures are notably smaller than in adults and standard resolution (around 1 mm) poses challenges in the confident assessment of anatomy and identification of relevant pathology.

Finally, PCD-CT offers the advantage of utilizing the spectral information of each X-ray photon, which overcomes the limitations of tissue characterization encountered with conventional EID-CT.^1^ This improved tissue characterization has applications in chest imaging, enabling the study of pulmonary perfusion as well as in musculoskeletal imaging for detecting bone marrow oedema and minimizing beam hardening metal artefacts.

Our hospital was among the first in Europe to install a PCD-CT scanner (Naeotom Alpha Siemens Healthineers, Erlangen, Germany) for clinical use in May 2021. After software and protocol optimization, the first paediatric examinations were conducted from January 2022. This pictorial review aims to present our clinical experience with PCD-CT in a selection of paediatric cases. Organized by anatomical region, from head to toe, the pictorial essay showcases several applications of PCD-CT through clinical examples.

### Head and neck

CT imaging of the brain in children finds application across diverse clinical scenarios, serving as an indispensable tool for diagnosis, monitoring and treatment strategies.^4^^,^^5^ Specifically, in the paediatric population, CT scans are regularly used to evaluate sequelae of head trauma; causes of particularly acute onset of symptoms; the presence of intracerebral pathology (ie, brain tumours, vascular abnormalities, cysts) and congenital anomalies. PCD-CT technology offers several advantages for brain imaging in children.

**Figure 1.**
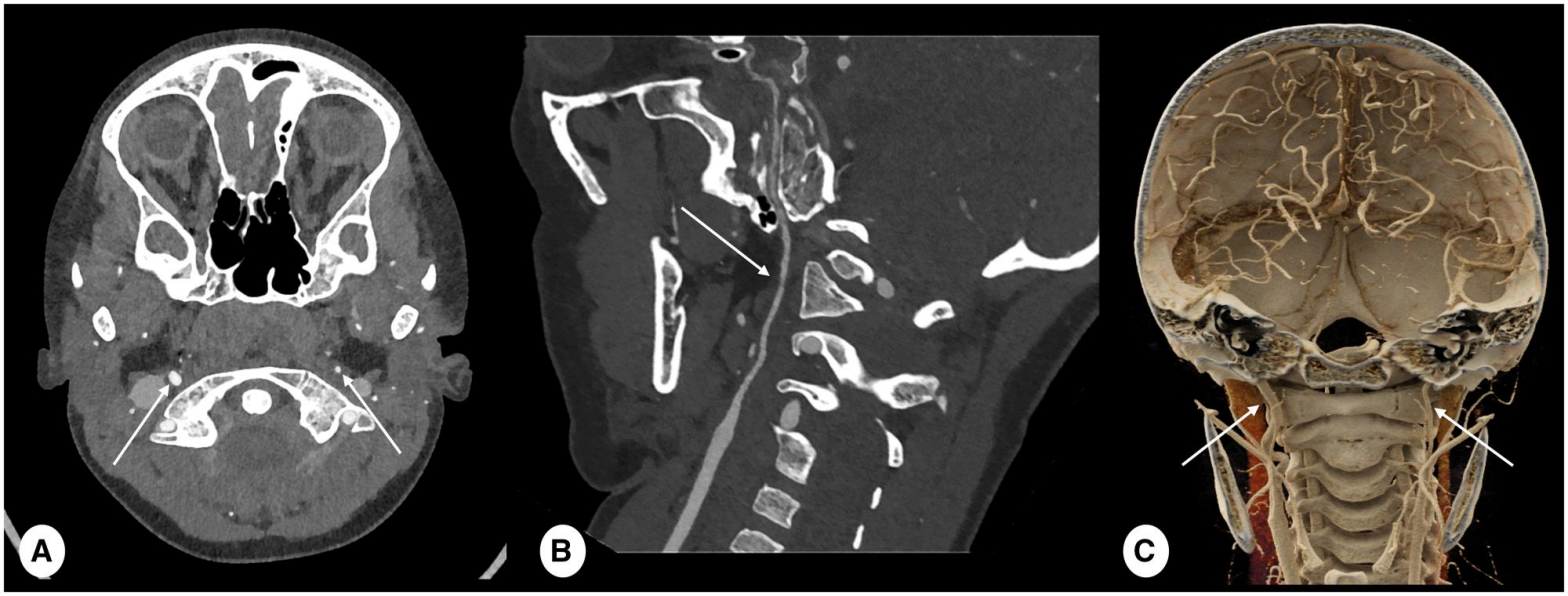
CT angiography (CTA) scan of a 7-year-old male with a hypoplastic carotid artery. (A) an axial ultra-high resolution (UHR) CTA is captured with a slice thickness of 0.2 mm, employing kernel Hv56 and iterative reconstruction (IR) level 4 (Q4). This image distinctly illustrates the disparity in size between the left hypoplastic internal carotid artery (ICA) (indicated by the thin arrow) and the right ICA (indicated by the thick arrow). (B) It corresponds to the curved planar reformat (CPR) along the central line of the left hypoplastic ICA (arrow). Meanwhile, (C) offers a coronal volume rendering of (A), accentuating the identical difference in calibre between the left hypoplastic ICA (thin arrow) and the right ICA (thick arrow). Note that the implementation of UHR scanning in combination with a reconstruction technique optimized for vascular structures, greatly enhances the crisp definition of vessel contours, significantly simplifying the assessment of vascular structures and enabling high quality multiplane reconstructions.

**Figure 2.**
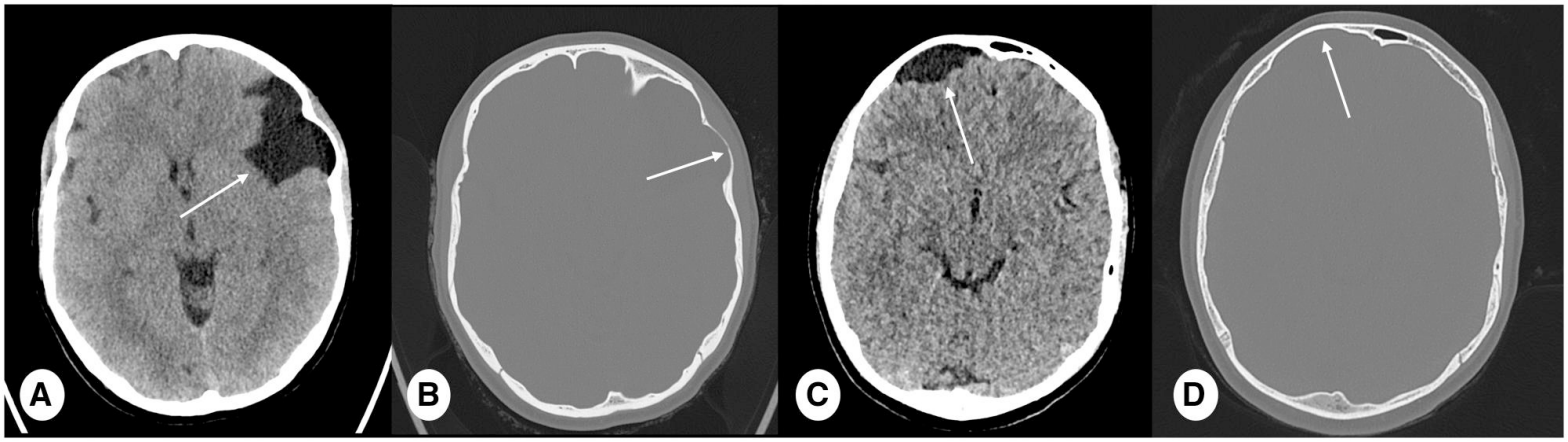
CT brain scan of a 17 year old female with arachnoid cyst (A, B). (A) PCD-CT ultra-high resolution CT (slice thickness 1 mm, kernel Hr40, and IR Q4) and (B) PCD-CT (slice thickness 1 mm, reconstruction kernel HR68, IR Q3). Corresponding EID-CT of a 12 year old male with arachnoid cyst in the brain (C, D). (C) Slice thickness 1 mm, kernel Hr40, IR level of 3, and (D) slice thickness 0.75 mm, kernel Hr60, and IR 3. (A) and (C) show an outline of arachnoid cysts in both patients, where the PCD-CT (A) image has a lower noise and sharper definition of the abnormality (arrows). Moreover, (B) and (D) (arrows) exhibit bone remodelling with thinning diploe bone wall. This comparison highlights the potential advantages of PCD-CT in achieving higher spatial resolution.

Ultra-high resolution (UHR) protocols obtained with PCD-CT are pivotal in the assessment of vascular pathology in children during CT angiography (CTA). UHR images provide sharper delineation of vessel contours, a critical aspect for the identification of congenital vascular anomalies (Figure 1). The higher SNR and resolution of PCD-CT facilitate the assessment of focal brain lesion, such as arachnoid cyst (Figure 2).

### Temporal bone imaging

**Figure 3.**
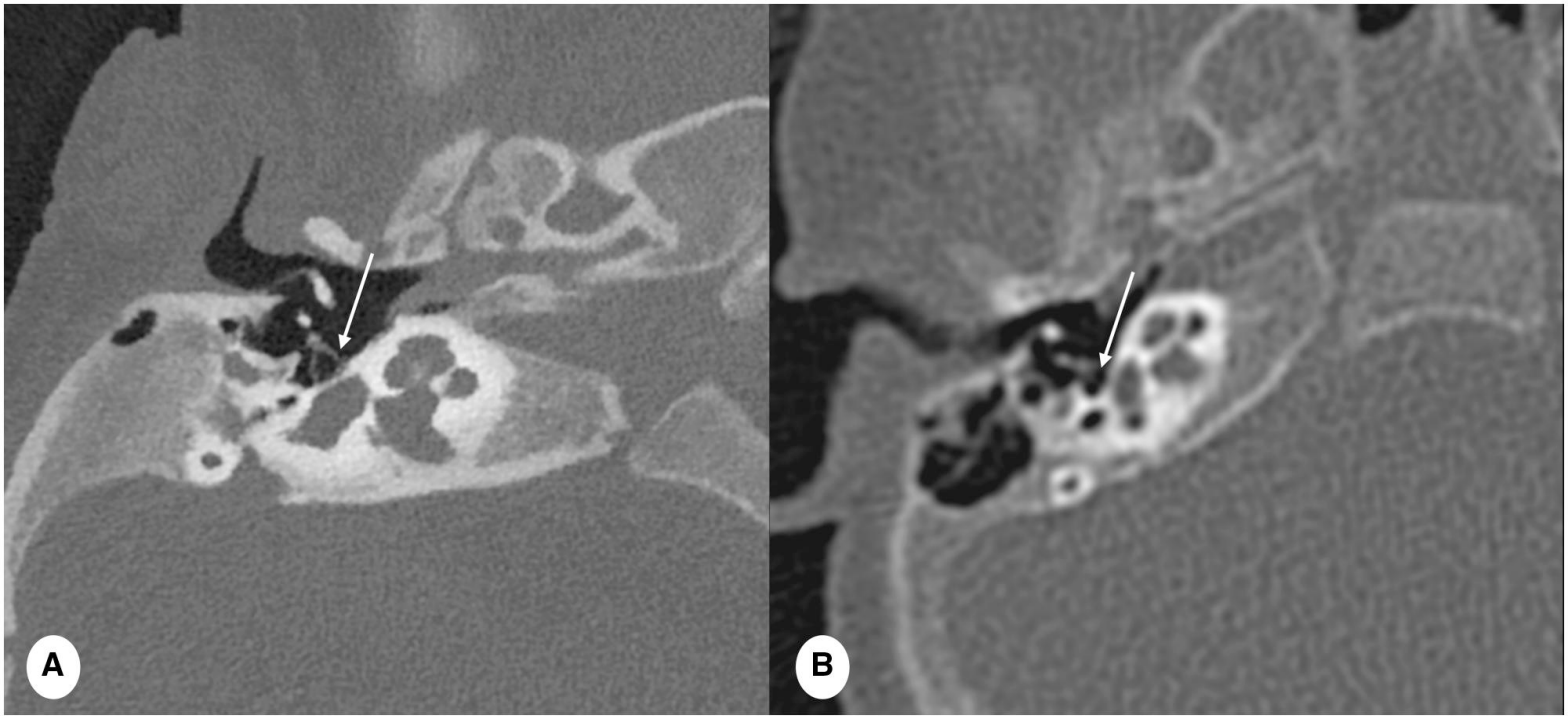
Comparison of temporal bone imaging performed with PCD-CT and EID-CT. PCD-CT scan in infant of 3 months, (A) slice thickness 0.2 mm, reconstruction kernel Hr84 and IR Q3 and EID-CT image in infants of 5 months, (B) slice thickness 0.6 mm, reconstruction kernel Hr56 and IR 3. Note the increase in image sharpness achieved by PCD-CT, clearly delineating the stapes bone (arrow in A) and the contours of the semicircular channels and cochlea. Conversely, EID-CT fails to visualize the stapes bone legs (arrow in B) and shows blurred contours of the internal ear’s structures. Noteworthy, the total dose-length-product for PCD-CT and EID-CT was 56 mGy×cm and 80 mGy×cm, respectively. Remarkably, despite the enhanced spatial resolution of the PDC-CT, these images were obtained at a lower dose compared to standard EID-CT.

CT is a frequently employed technique to evaluate middle ear pathology in children.^6^ High-quality images are necessary for detecting congenital anomalies that might underlie hearing loss in paediatric patients.^7^ Additionally, CT facilitates preoperative planning prior to cochlear implant surgery and postoperative evaluations. These applications hold particular significance in infants and young children, as early detection and treatment of hearing impairment is essential for normal development. PCD-CT brings a significant advancement in this anatomical area, which offers the potential to enhance temporal bone imaging sharpness. This contributes to superior image quality compared to the existing EID-CT, without incurring additional radiation dose (Figure 3).

### Nasal cavities imaging

**Figure 4.**
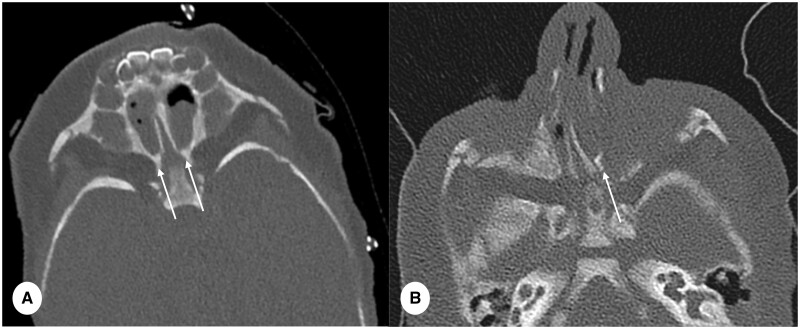
Comparison of CT sinuses for choanal atresia performed with PCD-CT and EID-CT. and a. (A) PCD-CT in a female of 23 days (slice thickness 0.6 mm, reconstruction kernel Hr72 and IR Q3) and (B) EID-CT image in a male of 4.5 months (slice thickness 0.75 mm, reconstruction kernel J70 and IR 3). Image (A) show bilateral bone choanal atresia (thin arrows) and image (B) shows unilateral left choanal atresia (thick arrow). It is important to observe the significantly improved signal-to-noise ratio and enhanced bone edge detection on (A), highlighting the advantages of PCD-CT over EID-CT in the assessment of choanal atresia in paediatric patients.

CT is a standard procedure for evaluating various nasal and sinus-related conditions in paediatric patients.^8^ These encompass a spectrum of pathologies, such as sinusitis, nasal polyps, and congenital anomalies, including choanal atresia (Figure 4). When it comes to assessing nasal cavity pathology, the increased spatial resolution and superior SNR offered by PCD-CT confer a significant advantage, enhancing the detection capabilities for these conditions.

### Thoracic imaging

**Figure 5.**
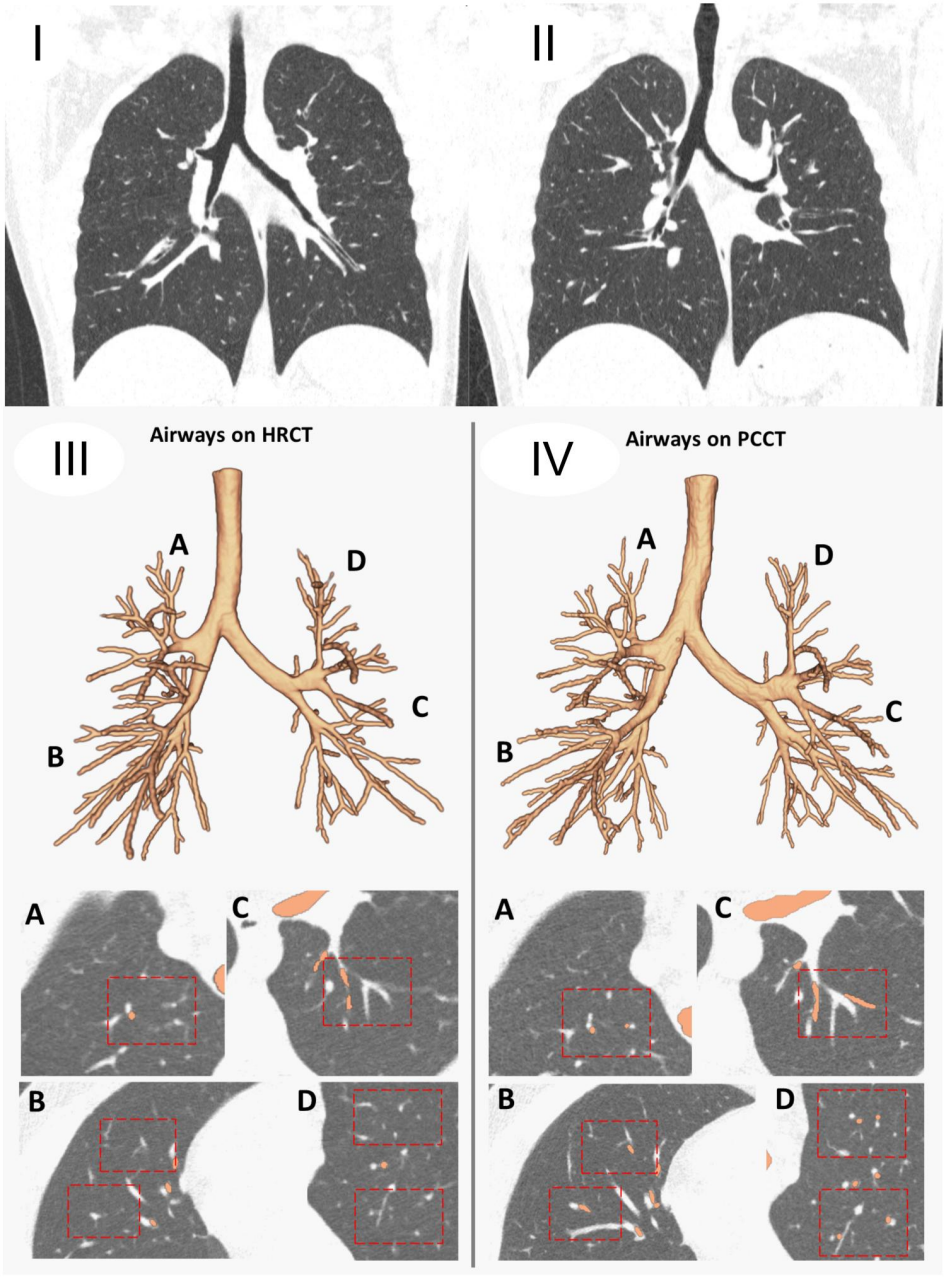
Effect of increased spatial resolution in peripheral airways detection in cystic fibrosis lung disease. Coronal (I) EID-CT image at 1 mm (reconstruction kernel Qr51 and IR 3) and (II) PCD-CT images at 1 mm (reconstruction kernel Qr56 and IR level 2, Q2). (III) and (IV) are the corresponding automatic airways segmentation obtained with artificial intelligence based software LungQ (Thirona B.V., Nijmegen, Netherlands) on the left side on the EID-CT and on the right side the PCD-CT. Noticeably when using the same spatial resolution (1 mm isotropic), Lung Q detected a higher number of airways in PCD-CT images compared to EID-CT images. Specifically 321 and 264 airway branches were identified in the respective scans. This reflects an increase of 57 branches (21.6%) in comparison to the EID-CT scan. Differences in airway branch detection are shown across 4 locations (A-D) in the image volume renderings, aligning with the identified areas on the CT images.

**Figure 6.**
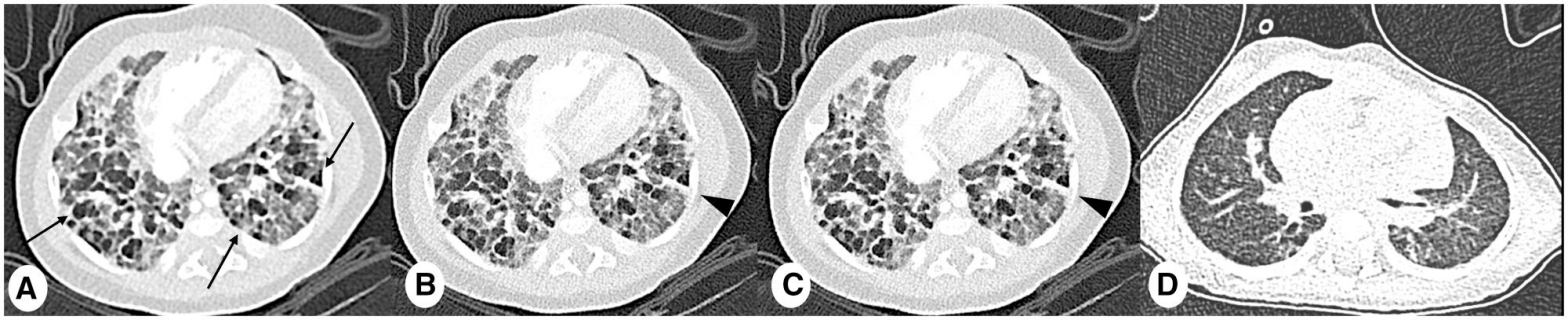
Comparison of image quality in infants with bronchopulmonary dysplasia (BPD). Axial PCD-CT images in 3 months old male with BPD, (A) slice thickness 1 mm, reconstruction kernel Bl56 and IR level 2, Q2, (B) slice thickness 1 mm, reconstruction kernel Bl64 and IR Q2, and (C) slice thickness 0.6 mm, reconstruction kernel Bl64 and IR Q2. (D) EID-CT coronal image (slice thickness 1 mm, reconstruction kernel Bl57 and IR 3). Note significant increase of sharpness and reduced noise from PCD-CT images, which facilitate assessment of typical BPD features, such as hypodense focal abnormalities (thin arrows) and linear fibrotic consolidations (arrowheads). Both scans are obtained with a dose-length product (DLP) of 4 mGy×cm.

**Figure 7.**
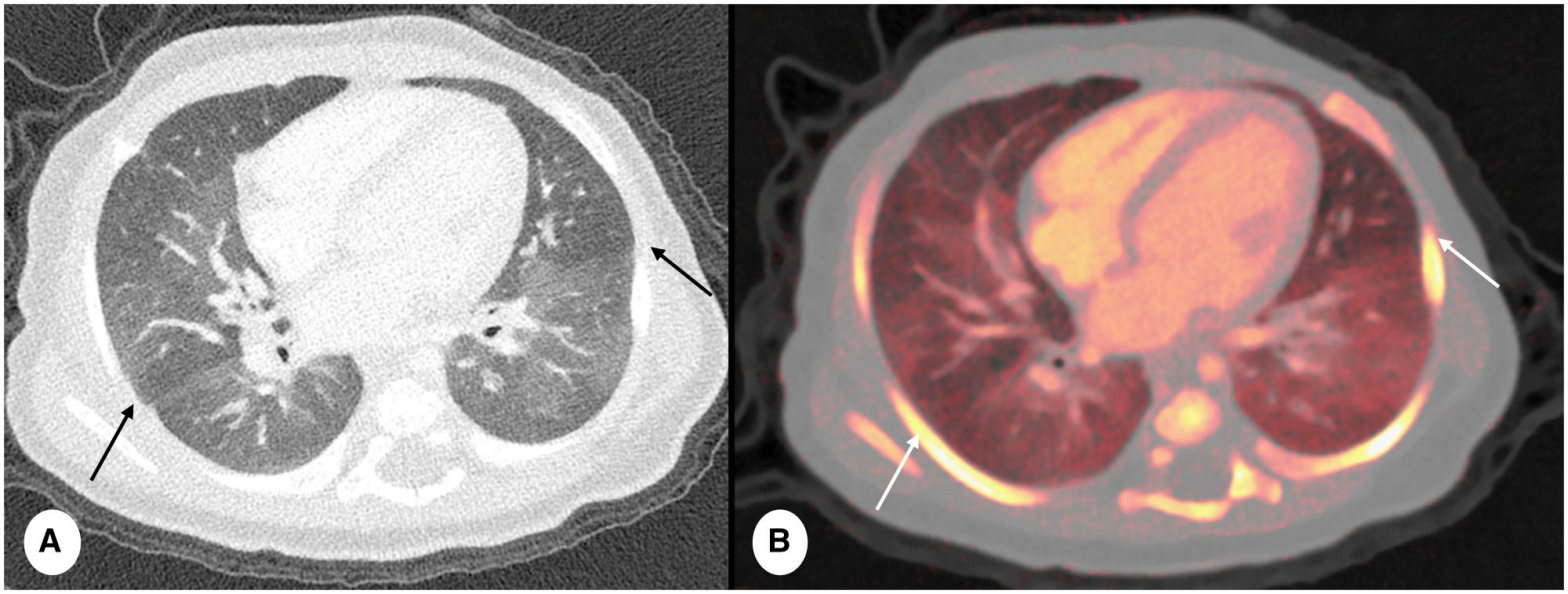
Example of PCD-CT spectral imaging in 3 months old female, premature born and persistent dyspnoea. (A) Axial free-breathing PCD-CT (slice thickness 0.6 mm, reconstruction kernel Qr64 and IR level 2, Q2) and (B) axial perfused blood volume (PBV) reconstruction, slice thickness 2 mm. PBV reconstructions provide a comprehensive visualization and quantitative assessment of iodine uptake within the lung parenchyma. This imaging technique allows for the precise depiction of iodine attenuation at a singular time point, effectively serving as a surrogate marker for lung perfusion. Note the mosaic pattern observed within the lung parenchyma in (A) (arrows) that match areas exhibiting reduced iodine density also correspond to diminished iodine uptake in the PBV reconstruction (arrows in B).

CT is the current gold standard to assess pulmonary disease in children.^9^ PCD-CT emerges as a game-changer in pulmonary imaging, especially for children under the age of 5, where PCD-CT can increase visualization of peripheral airways, bronchial wall, small cystic lesions. The improved image quality is likely to results in an increased sensitivity to detect disease progression in chronic lung diseases, such as cystic fibrosis (Figure 5). An example of this was recently demonstrated in adult, in the long term follow-up of interstitial lung disease.^10^ With increased spatial resolution and the integration of artificial intelligence tools, PCD-CT is set to revolutionize the identification of peripheral airway abnormalities (Figure 5) and parenchymal disorders. In the case of younger children, these sharper images will instil greater confidence among radiologists, facilitating the diagnosis of complex conditions, such as interstitial lung diseases (Figure 6). Finally, PCD-CT is capable of iodinated contrast agent identification, which facilitates virtual non-contrast imaging and perfused blood volume (PBV) quantification. These advanced techniques can provide a comprehensive assessment of ventilation and perfusion issues in a single examination, as exemplified in (Figure 7) and hold the promise of opening completely new diagnostic approaches.^11^

### Cardiovascular imaging

The current commercially available PCD-CT scanner is a dual-source system providing high temporal resolution which is vital for cardiac imaging. In addition, the high pitch scan mode allows for imaging very young children without the need for anaesthesia. Contrast timing is difficult in complex cardiac pathology. The use of mono-energetic imaging may boost the visibility of contrast material in case of suboptimal enhancement due to contrast timing problems.

**Figure 8.**
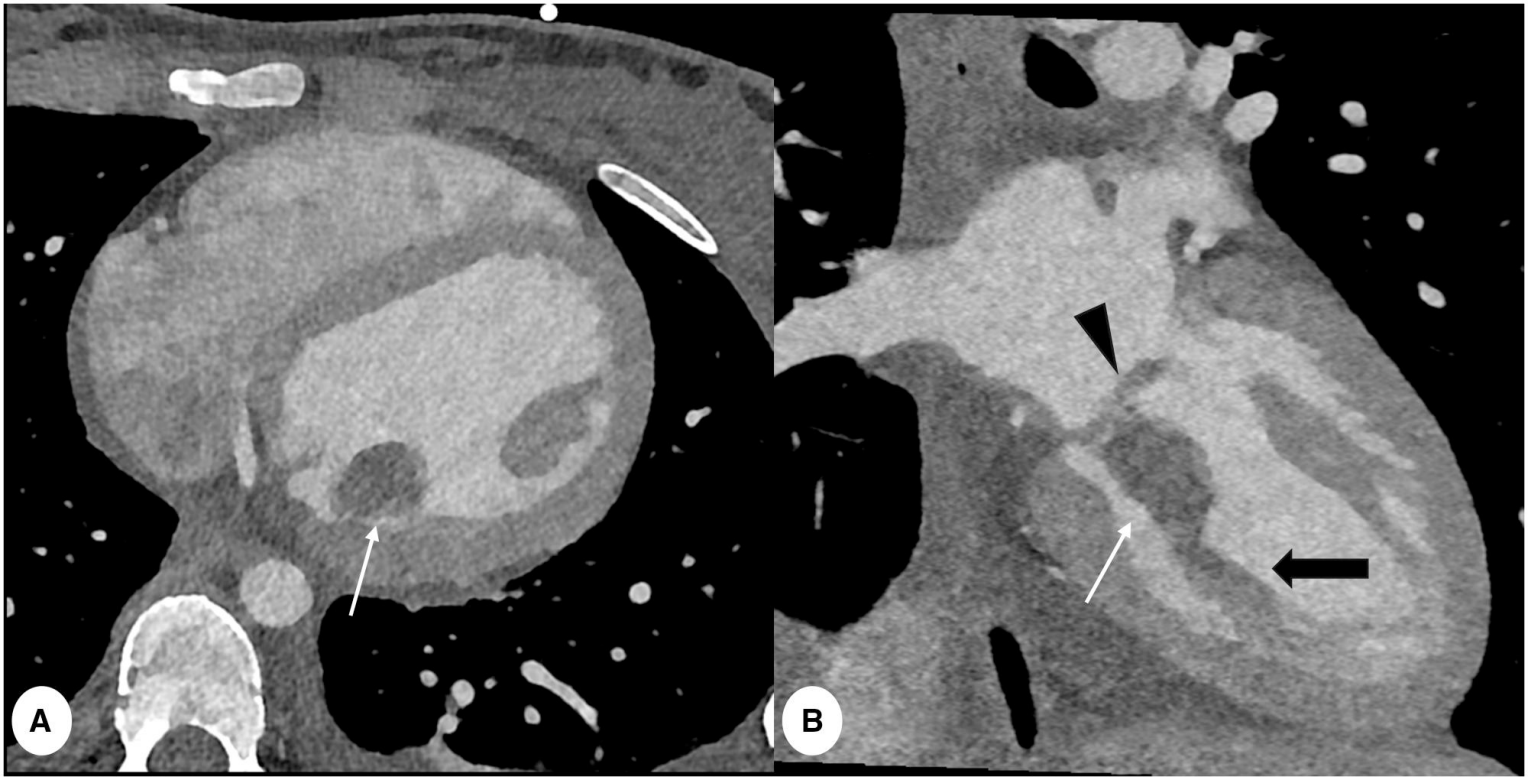
Electrocardiogram (ECG) triggered cardiac CT in an 18 years female with suspicious septic endocarditis and multiple embolism by S. *Aureus* bacteraemia. (A) Axial and (B) coronal PCD-CT (slice thickness 0.4 mm, reconstruction kernel Bv48 and IR level 4, Q4), shows a hypodense lesion (white arrows) on the mitral valve (arrowhead in B) and on the papillary muscles (thick arrow in B) of the mitral valve. Note sharpness of the images which allows clear visualization of the mitral cusps (arrowhead).

Thus, PCD-CT offers better cardiovascular imaging quality at a similar radiation dose compared to EID-CT in infants with suspected cardiac heart defects.^12^ The increased sharpness of PCD-CT provides greater anatomical detail than EID-CT allowing for clear definition of intra-cardiac lesions (Figure 8).

### Musculoskeletal imaging

**Figure 9.**
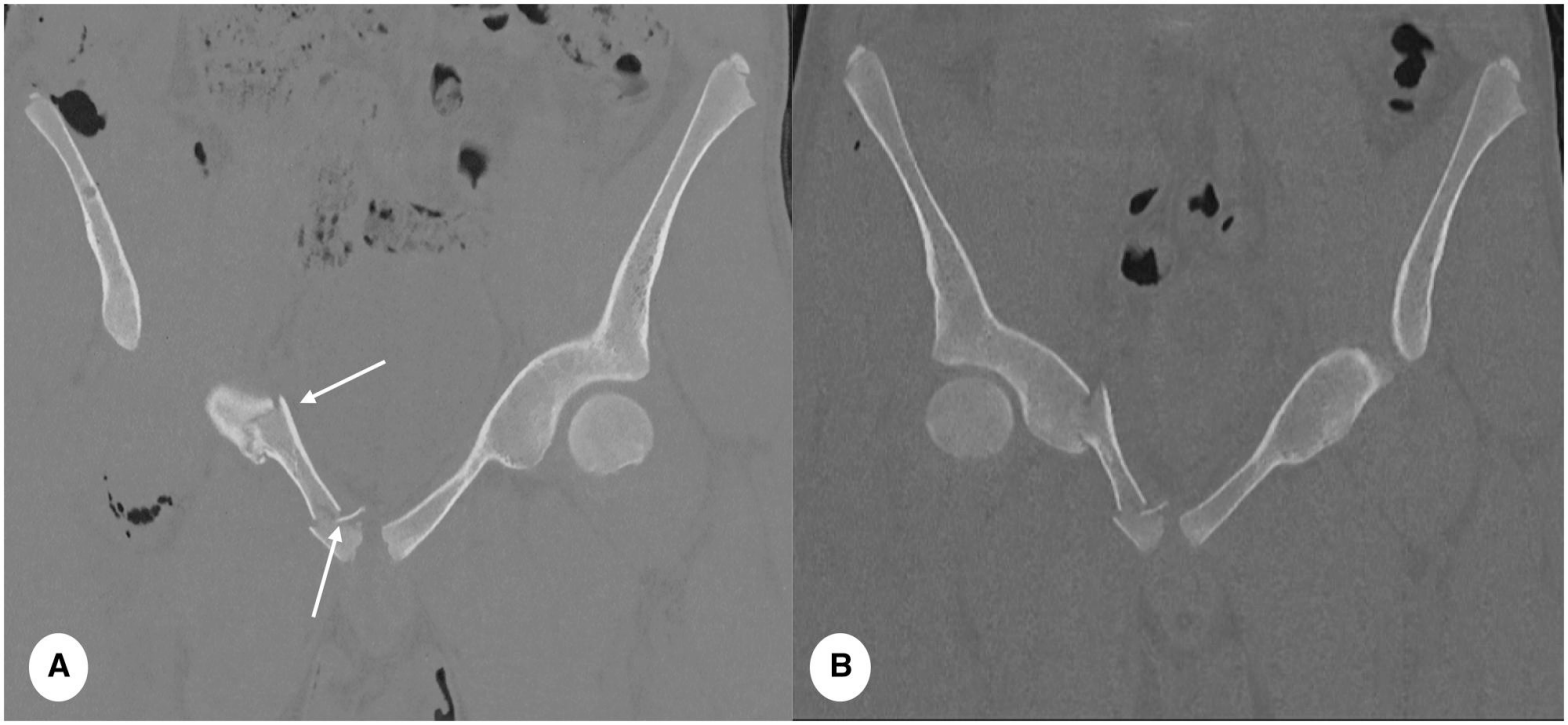
Comparison of hip fracture assessment between PCD-CT and EID-CT in 16 year old male after pelvic trauma. (A) PCD-CT (slice thickness 1 mm, reconstruction kernel Br68 and IR level 3, Q3) and (B) EID-CT (slice thickness 1 mm, reconstruction kernel l70h and IR 3) on the same subject. Note higher SNR and clearer definition of cortical bone fracture fragments (thin arrows) in (A) and superior pubic ramus fracture (thick arrow). Bone contours in (B) are less sharp with reduced signal of cortical bone.

**Figure 10.**
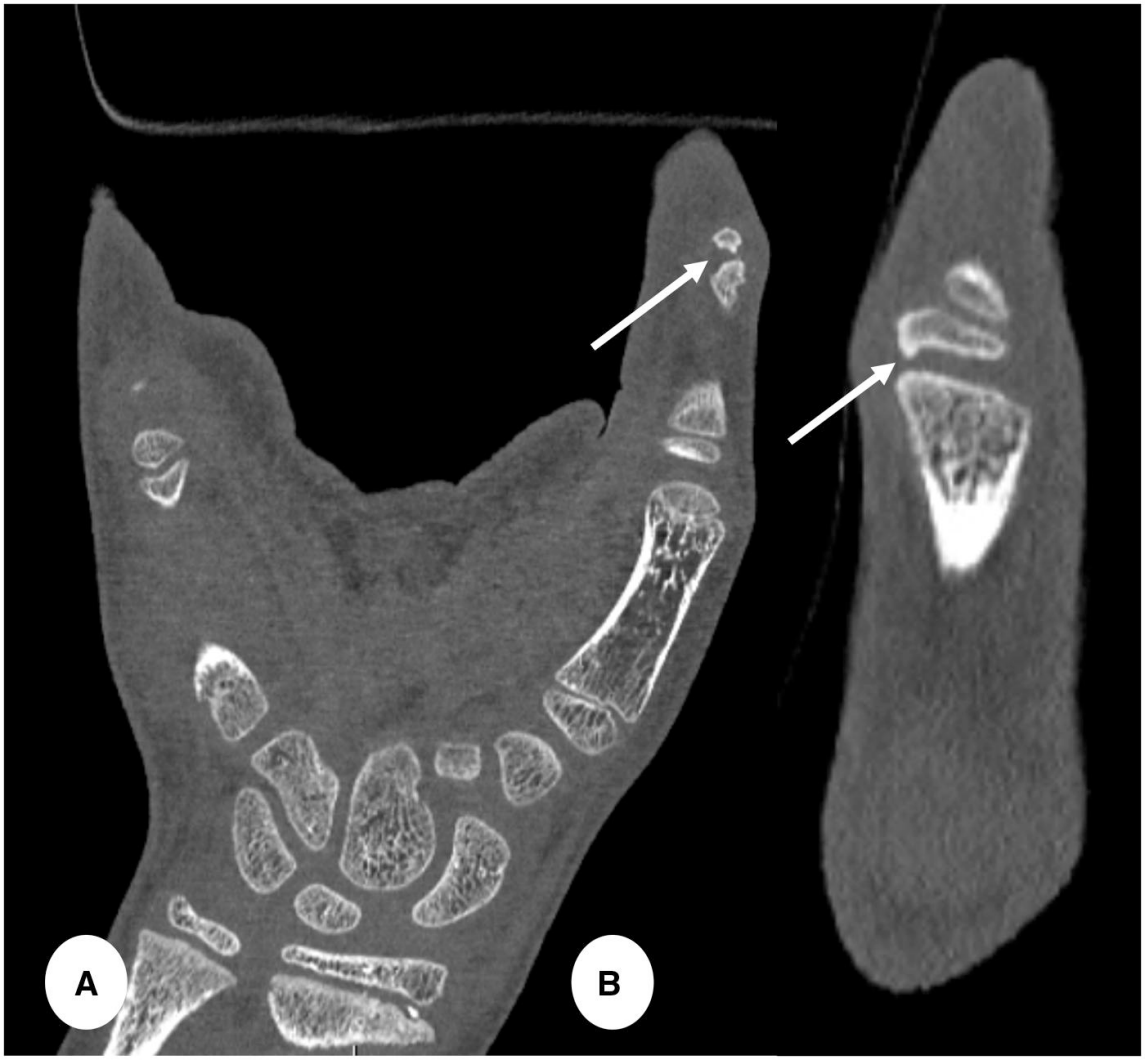
Hand CT in 10 years old male with swelling and pain proximal interphalangeal (PIP) joint of the left thumb from Trevor disease. (A) Coronal and (B) magnified sagittal PCD-CT images (slice thickness 0.2 mm, reconstruction kernel Br89 and IR level 3, Q3) showing small exophytic bone apposition (arrows in A and B) at the level of the proximal epiphysis of the PIP joint of the left thumb, likely representing small osteochondroma.

**Figure 11.**
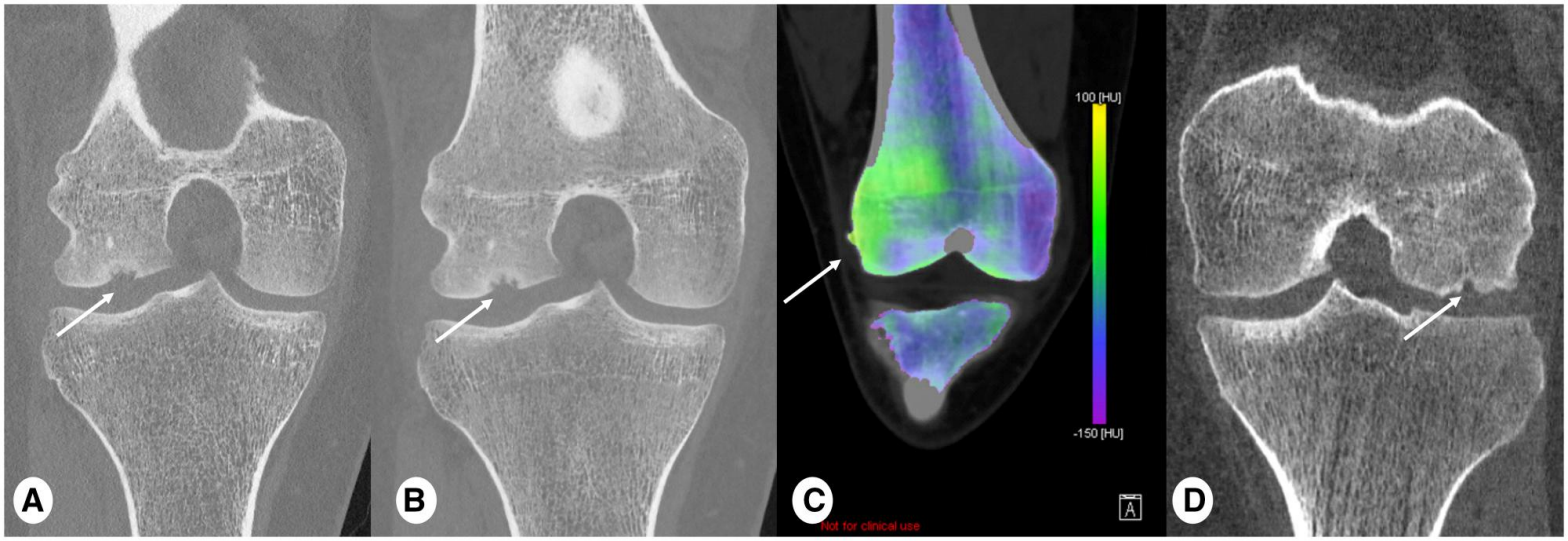
Comparison PCD-CT and EID-CT to assess osteochondritis dissecans (OCD). (A) 0.2 mm and (B) 0.6 mm coronal PCD-CT images (reconstruction kernel Br89 and IR level 3, Q3) and (C) Virtual Non-Calcium (VNCa) imaging for bone marrow oedema detection 3 mm multiplanar reformat in a 16 years old male showing cortical defect at the lateral femoral condyle of the right knee (arrows in A and B) after sport trauma. (D) Coronal EID-CT (slice thickness 0.6 mm, reconstruction kernel Br89 and IR 3, Q3) in a 16 years old male showing a cortical defect at lateral femoral condyle of the left knee (arrow). Note clear definition of the cortical contours of OCD lesion on image (A) and (B) and reduced noise at the same slice thickness ((B) vs. (D)). Interestingly, total dose-length product (DLP) was 200 mGy×cm and 522 mGy×cm for PCD-CT and EID-CT, respectively. Colour maps in (B) is showing higher density of lateral femoral condyle of the right knee (green colour/arrow) due to higher water content for bone marrow oedema. VNCa reconstruction for bone marrow oedema are still provided as non-clinical/research tool on Naeotom Alpha scanner (Siemens Healthineers, Erlangen, Germany).

**Figure 12.**
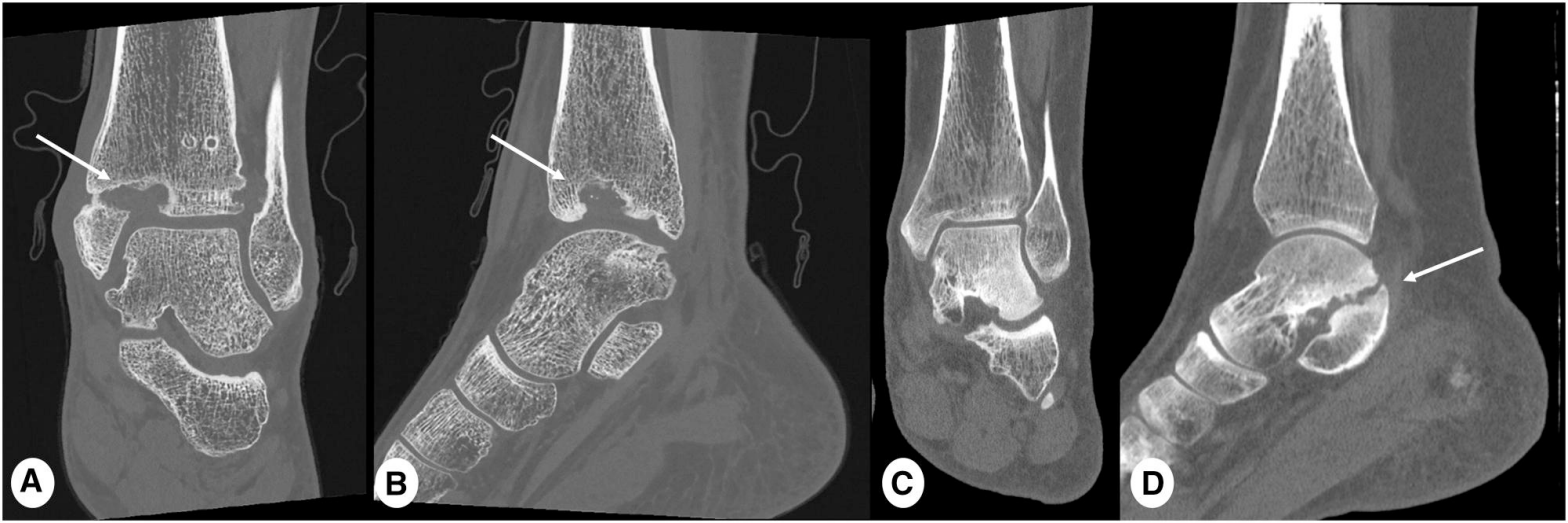
Example of assessment of osteomyelitis. (A) Coronal and (B) sagittal multiplanar reformat PCD-CT images (slice thickness 0.6 mm, reconstruction kernel Br76 and IR level 3, Q3) in 18 years old male with large tibial bone defect (arrows) in the distal left tibia extending to the medial malleolus caused by osteomyelitis. (C) Coronal and (D) sagittal EID-CT images reformats (slice thickness 0.75 mm, reconstruction kernel Ur77 and IR 3) in 18 years old male after surgical correction of talocalcaneal coalition (thick arrow). Note increase spatial resolution and definition of bone contours and spongiosa in PCD-CT image compared to EID-CT image. Moreover total radiation exposure was lower in PCD-CT than EID-CT with total dose-length product (DLP) was 75 mGy×cm and 327 mGy×cm for PCD-CT and EID-CT, respectively.

**Figure 13.**
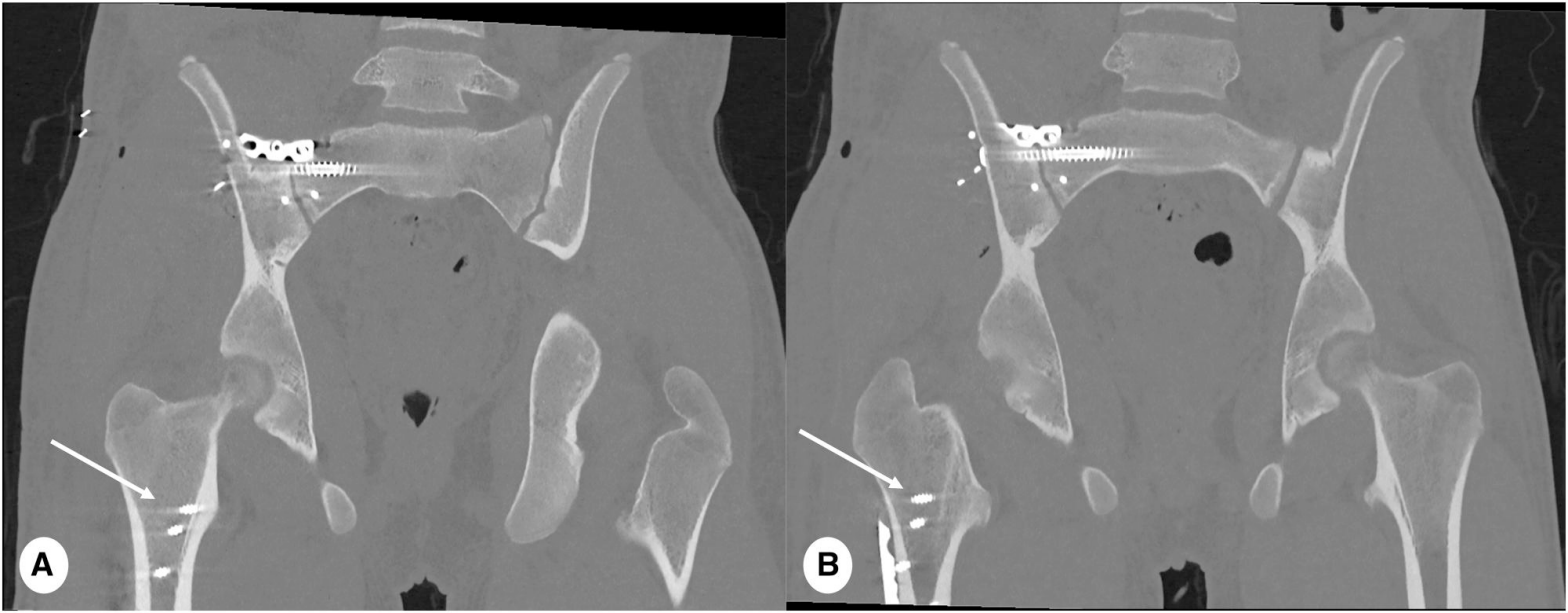
CT Pelvis with metal artefact reduction technique in 16 years old male after surgery for multiple traumatic pelvic bone fractures. (A) Coronal 80 kiloelectronvolt (keV) and (B) 110 keV metal reduction multiplanar reformats PCD-CT images (slice thickness 1 mm, reconstruction kernel Br72 and IR level 3, Q3). Note that higher keV reconstruction allows to reduce blooming artefact from metal implants (arrows in A and B). This is achieved through spectral imaging techniques, which by adjusting the keV settings, effectively reduce metal artefacts. The energy level in keV refers to the specific single energy level retrospectively selected for the reconstruction of virtual mono-energetic images.

CT is routinely used in paediatric musculoskeletal disease evaluation and diagnosis.^13^ Common applications encompass the assessment of fractures (Figure 9), bone tumours (Figure 10), osteochondritis dissecans (OCD) (Figure 11), and osteomyelitis (Figure 12). The advanced capabilities of PCD-CT stand out in this context, offering higher spatial resolution. This enhanced resolution allows for highly detailed visualization of skeletal structures, including exact demarcation of the cortical and trabecular bone.^14^ Furthermore, PCD-CT's spectral imaging enables the classification of specific tissue characteristics, such as the identification of bone marrow oedema (Figure 11C). By employing Virtual Non-Calcium imaging, it becomes possible to subtract the high-attenuating calcium signal from a fracture or at OCD site, thereby detecting the presence of bone marrow oedema. Additionally, PCD-CT proves invaluable in mitigating beam hardening artefacts in post-surgery patients after metal implant (Figure 13), ensuring clearer and more accurate postoperative assessments.

### Abdominal imaging

**Figure 14.**
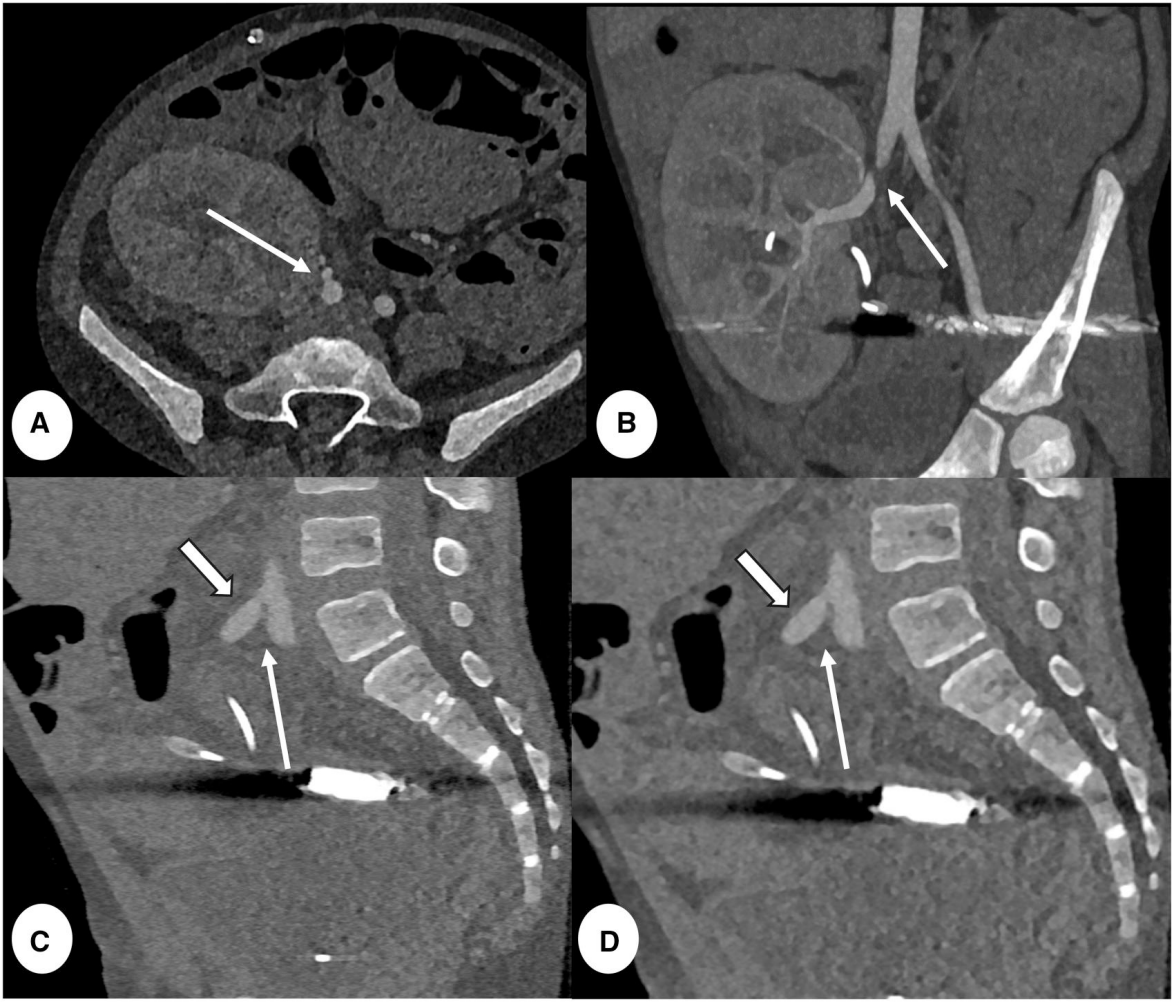
CT angiography abdomen in 9 years old male after kidney transplant complicated by renal artery anastomosis stenosis. (A) 0.2 mm axial, (C) 0.4 mm sagittal and (D) 0.6 mm sagittal PCD-CT images (reconstruction kernel Bv48 and IR level 4, Q4). (B) A 6 mm curved maximum intensity projection (MIP). Note lumen stenosis at the level of the anastomosis with the right common iliac artery (thin arrow A-D) and post-stenotic renal artery dilatation (thick arrow in C and D). CTA was conducted with a total dose-length-product (DLP) of 7 mGy×cm.

**Figure 15.**
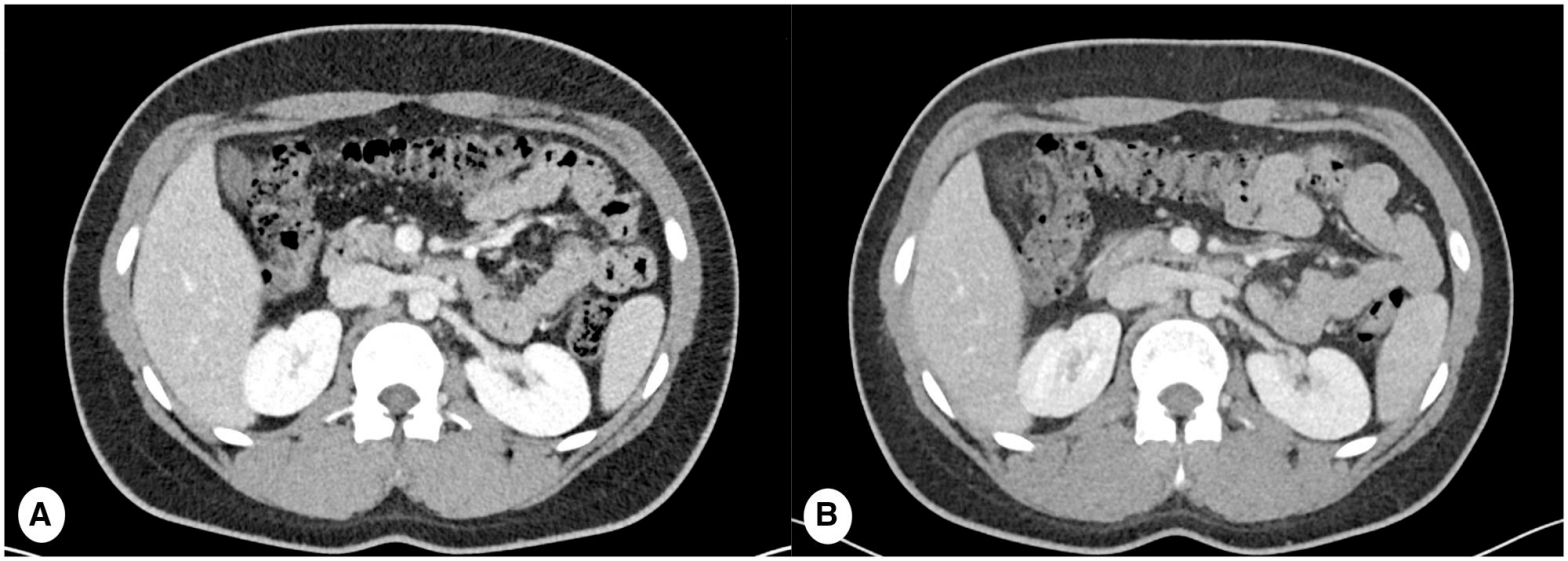
Comparison of abdominal imaging in the same patient scanned for Testicular seminoma. (A) PCD-CT scan and (B) EID-CT, both conducted with a uniform window width (WW) and window level (WL) set at 400 and 40, respectively. Both images had a slice thickness of 3 mm, reconstruction kernel Br40, IR Q3. The PCD-CT images exhibit a notably higher Signal-to-Noise Ratio (SNR) compared to the EID-CT image as it showcases enhanced contrast at the level of the kidneys. PCD-CT also provides sharper delineation of organ contours and blood vessels.

Paediatric abdominal CT scans deliver high-resolution cross-sectional images crucial for diagnosing and assessing a range of conditions in children.^15^ These conditions encompass acute causes of abdominal pain, such as appendicitis, trauma-related injuries, the presence of tumours, and congenital anomalies. Also for abdominal imaging, PCD-CT offers a significant advantage by enhancing spatial resolution and SNR, thus ensuring exceptional image quality, even when evaluating vascular pathologies (Figure 14) and abdominal anatomy (Figure 15).

## Conclusions

PCD-CT presents the potential to transform current paediatric radiology practices.^16^ The substantial increase in spatial resolution and reduction in image noise yield exceptional image quality, enhancing sensitivity for pathology detection, especially in children below the age of 5. In this age group due to the small size of anatomical structures obtaining diagnostic image quality often necessitates raising radiation doses. For older children requiring repeated imaging over time (ie, cystic fibrosis) where standard spatial resolution (typically around 1 mm) suffices for diagnosis, PCD-CT still offers clear advantages. Its higher SNR can potentially enable further reductions in CT radiation doses.

Moreover, the introduction of spectral imaging capabilities in PCD-CT, coupled with the UHR mode, paves the way for exploring novel clinical applications. This includes the assessment of cardiovascular anomalies, pulmonary perfusion, and bone marrow oedema, offering a broader spectrum of diagnostic possibilities.

The images featured in this pictorial review provide only a glimpse of the immense PCD-CT potential in the clinical setting of paediatric imaging (Table 1).

## Figures and Tables

**Table 1.** Summary of possible photon counting CT applications in paediatric population.

Applications of PCD-CT in paediatric imaging	Advantages compared to EID-CT
General advantages	Higher resolution at lower dose Safer repeated imaging at lower dose Dose reduction of iodine contrast Spectral imaging capabilities
Head imaging	Higher resolution and SNR for angiographic studies, allowing better assessment of vascular pathology Improved assessment of craniosynostosis Radiation dose reduction for specific indications, such as assessment of ventricular drain/hydrocephalus in non-collaborative children
ENT imaging	Increased spatial resolution and SNR for temporal bone imaging, aiding in the assessment of middle and internal ear abnormalities Reduction of metal artefacts for better evaluation of partial or total ossicular replacement prostheses (PORP and TORP) and cochlear implants Enhanced visualization of bone structures for assessment of nasal cavities pathology Enhanced differentiation of soft tissue
Chest/Thorax imaging	Enhanced image quality in paediatric pulmonary pathology, especially in children below 5 years Improved visualization of peripheral airways, bronchial wall and bronchial segmentation (e.g. bronchial atresia assessment) Improved visualization of small cystic lesion (subpleural and intraparenchymal) Spectral imaging: virtual non-contrast (VNC), pulmonary blood volume (PBV) High resolution imaging of vascular anomalies and aberrant vessels
Cardiovascular imaging	Higher spatial resolution of cardiovascular anomalies Reduction of metal artefact with spectral imaging (high keV) Better stent lumen imaging Spectral imaging: pure lumen imaging
Musculoskeletal imaging	Better assessment of complication in orthopaedic surgery (metal artefact reduction) Improved visualization and detection of fractures and bone-implant interfaces Improved bone assessment and bone healing processes Spectral imaging for bone marrow oedema: virtual non calcium (VNCa) imaging
Abdominal imaging	Better differentiation between bowel wall and mesenteric fat Better bowel wall contrast enhancement assessment (e.g. in inflammatory bowel disease) Enhanced visualization of vascular structures (e.g. renal artery stenosis)
